# EGFR amplification indicated poor prognosis in EGFR‐mutated lung cancer with leptomeningeal metastases

**DOI:** 10.1111/crj.13733

**Published:** 2024-02-01

**Authors:** Di Geng, Ruina Niu, Jinghong Li, Sanxing Guo, Qianqian Guo, Siyuan Huang, Yurong Wang

**Affiliations:** ^1^ Department of Medical Oncology The First Affiliated Hospital of Zhengzhou University Zhengzhou China; ^2^ Department of Neurology The First Affiliated Hospital of Zhengzhou University Zhengzhou China; ^3^ Department of Neurology Laboratory The First Affiliated Hospital of Zhengzhou University Zhengzhou China

**Keywords:** cerebrospinal fluid, EGFR mutation, leptomeningeal metastases, lung cancer, next‐generation sequencing

## Abstract

**Background:**

Leptomeningeal metastasis (LM) is a lethal complication of advanced lung cancer, and due to limited access to the leptomeningeal lesion, we explored the potential role of cerebrospinal fluid (CSF) as a source for liquid biopsy in LM patients of lung cancer with EGFR mutation.

**Materials and Methods:**

From August 2018 to June 2021, we collected CSF samples of lung cancer patients at First Affiliated Hospital of Zhengzhou University. Next‐generation sequencing was performed to detect the mutations in EGFR genes, and 38 patients detected with EGFR mutations were finally enrolled in further clinical analyses.

**Results:**

TP53 missense mutation (50%) was the most frequently detected concurrent gene in CSF. In those 10 patients whose CSF was obtained upon resistance to first TKI, TP53 missense mutation (40%, *n* = 4) and EGFR copy number amplification (40%, *n* = 4) was detected with high frequency; meanwhile, T790M mutation was found only in two patients. Known mechanisms of acquired resistance to third EGFR‐TKIs were found in 31.8% of cases. C797S or C797G mutation was identified in three patients. Possible EGFR‐independent resistant mechanism included MET amplification (4.5%), RET gene fusion (4.5%), PIK3CA missense mutation (4.5%) and CDK4 amplification (4.5%). The median OS was 55 months, the median OS_LM_ was 38 months. EGFR amplification was associated with shortened OS in these EGFR‐mutated lung cancer with leptomeningeal metastases.

**Conclusion:**

EGFR amplification indicated poor prognosis in EGFR‐mutated lung cancer with leptomeningeal metastases, providing a novel pathogenesis and treatment direction of these patients.

## Graphical abstract

By NGS analysis of CSF, EGFR amplification was associated with shortened OS in these lung cancer with EGFR‐mutated lung cancer with leptomeningeal metastases.

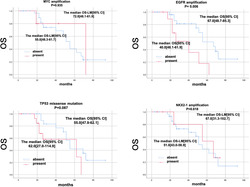

## 1 INTRODUCTION

An increased occurrence of 3%–5% in NSCLC with leptomeningeal metastasis (LM) has been observed since the prolonged OS of lung cancer patients.^1^, ^2^ Especially in EGFR‐mutated NSCLC, the incidence of LM was as high as 9%–9.4%.^2^ Up to now, LM is still a devastating complication, with a median survival time of 3–11 months despite advances in anticancer treatment.^2^, ^3^ Central nervous system (CNS) metastases harbour different resistance mechanisms,^4^, ^5^ so it is crucial to perform intracranial biopsy or re‐biopsy to test acquired resistance at CNS progression. In spite of the importance of biopsy and re‐biopsy to understand the genetic status and changes of tumour tissues, it is especially limited in CNS metastases including LM. The circulating cell‐free DNA (cfDNA) of plasma has been applied to predict clinical outcomes in NSCLC with dynamic changes of EGFR mutation status.^6^ As for brain tumour especially leptomeningeal metastasis, CSF plays a more important role than plasma in detecting cfDNA and indicating the unique genotyping.^4^, ^7^ However, only limited number of patients were included in these studies, and little is known about the resistance mechanisms of LM in lung cancer harbouring EGFR mutations.^8^, ^9^

In this study, we try to explore CSF in revealing clinically targetable genomic alterations and resistance mechanisms of LM in EGFR‐rearranged lung cancer, as meanwhile analyses the prognostic implications of concurrent mutation.

## 2 METHODS

### 2.1 Study population

From August 2018 to June 2021, at the First Affiliated Hospital of Zhengzhou University, 139 lung patients were included in this retrospective study. The diagnostic criteria of leptomeningeal metastasis were detecting of malignant cells by CSF cytology and/or typical findings of imageological examination (leptomeningeal enhancement or ventricle broadening). Among these 139 patients, CSF was available for NGS in 43 patients. Thirty‐eight patients were detected with EGFR mutation.

**FIGURE 1:**
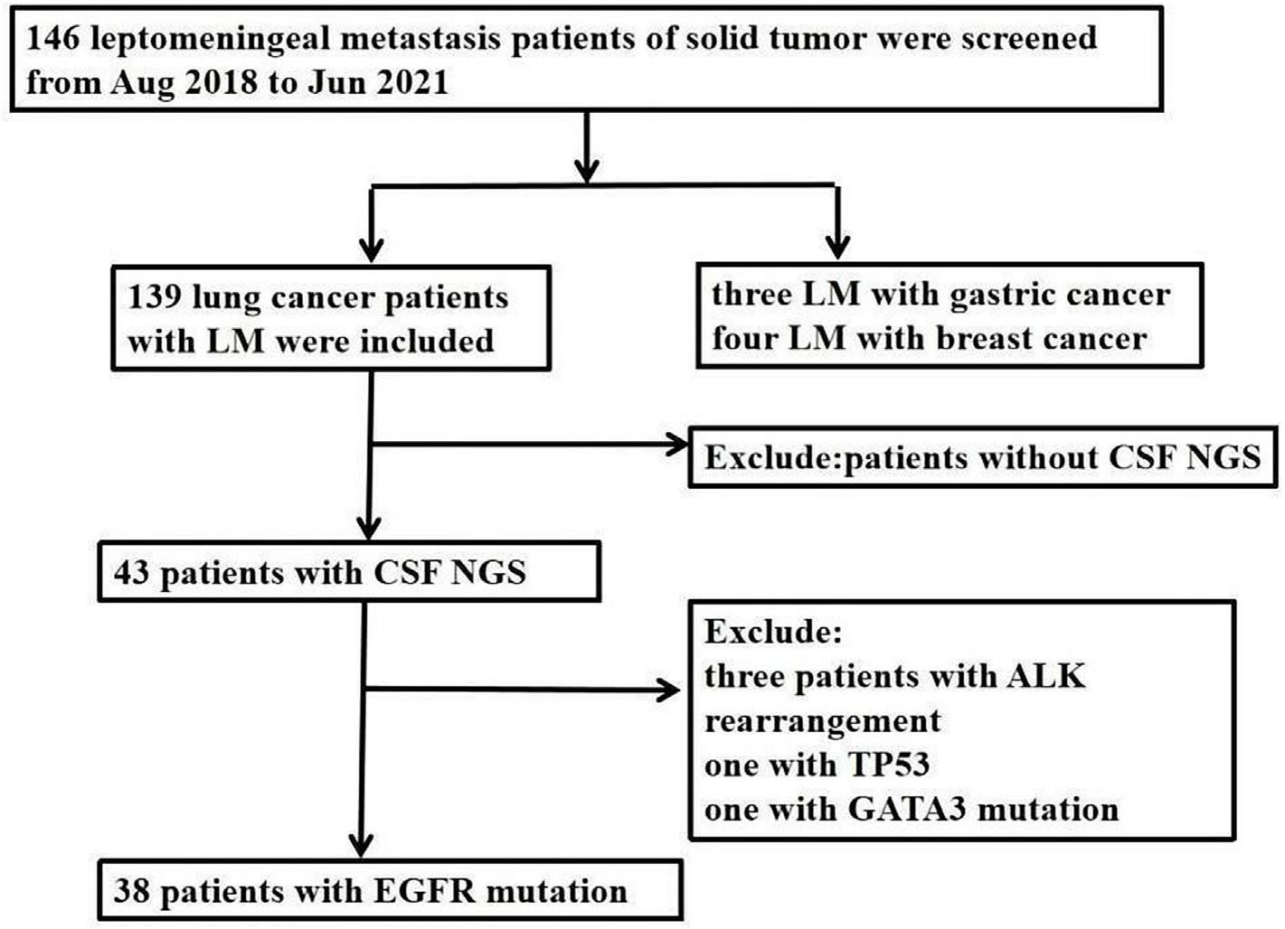
Study flow chart. ALK, anaplastic lymphoma kinase; CSF, cerebrospinal fluid; EGFR, epidermal growth factor receptor; LM, leptomeningeal metastasis; NGS, next generation sequencing.

Approximately 10 mL of CSF was collected by lumbar puncture for cytologic examination and NGS. Next‐generation sequencing targeting hotpot mutations in 425 genes was performed. And all the sampling and experimental protocols have been approved by the Research Ethics Committee of the First Affiliated Hospital of Zhengzhou University (Figure 1).

### 2.2 Statistical analysis

All the statistical analyses were performed using SPSS® software, version 25 (IBM Corp, Armonk, NY, USA). OS of leptomeningeal metastasis (OS_LM_) was defined as the time from LM diagnosis to the time of last follow‐up or death. OS and OS_LM_ were estimated using the Kaplan–Meier method. The differences between groups were analysed by the log‐rank test, and the value *p* < 0.05 was considered significant.

## 3 RESULTS

### 3.1 Patient characteristics

This study enrolled 38 lung cancer patients (14 men and 24 women) who met the inclusion criteria with LM. EGFR mutations of CSF cfDNA were detected in all 38 patients. The median age was 53 years (range between 30 and 72 years), and 36 patients (94.7%) were diagnosed with adenocarcinoma. Malignant cells were found in the CSF of 33 patients (86.8%), and 19 patients (50%) had typical imaging for LM. The median KPS score at the diagnosis of LM was 50. The clinical characteristics of these patients were summarised in Table 1. CSF of six patients was collected before they received EGFR‐TKI. CSF of 10 patients was obtained after failure to first TKI (icotinib = 4, gefitinib = 4, and erlotinib = 2). CSF of 22 patients was collected after failure on third TKI (almonertinib = 3 and osimertinib = 19).

**TABLE 1:** Patient demographic and baseline characteristics (*n* = 38).

	No. of patients (%) patients (%)
Age at the time of LM diagnosis (years)	
Median (range)	53 (30–72)
<60	31 (81.6%)
≥60	7 (18.4%)
KPS at the time of LM diagnosis	
Median (range)	50 (20–90)
≥60	23 (60.5%)
<60	15 (39.5%)
Gender	
Male	14 (36.8%)
Female	24 (63.2%)
Histologic subtype	
Adenocarcinoma	36 (94.7%)
Small cell lung cancer	1 (2.6%)
Undefined pathology	1 (2.6%)
Smoking status	
Current/former smoker	37 (97.4%)
Non‐smoker	1 (2.6%)
Diagnosis of LM	
Positive CSF cytology	33 (86.8%)
Typical brain imaging	19 (50%)

### 3.2 Gene profiles presented by CSF NGS

#### 3.2.1 EGFR detection in CSF

**FIGURE 2:**
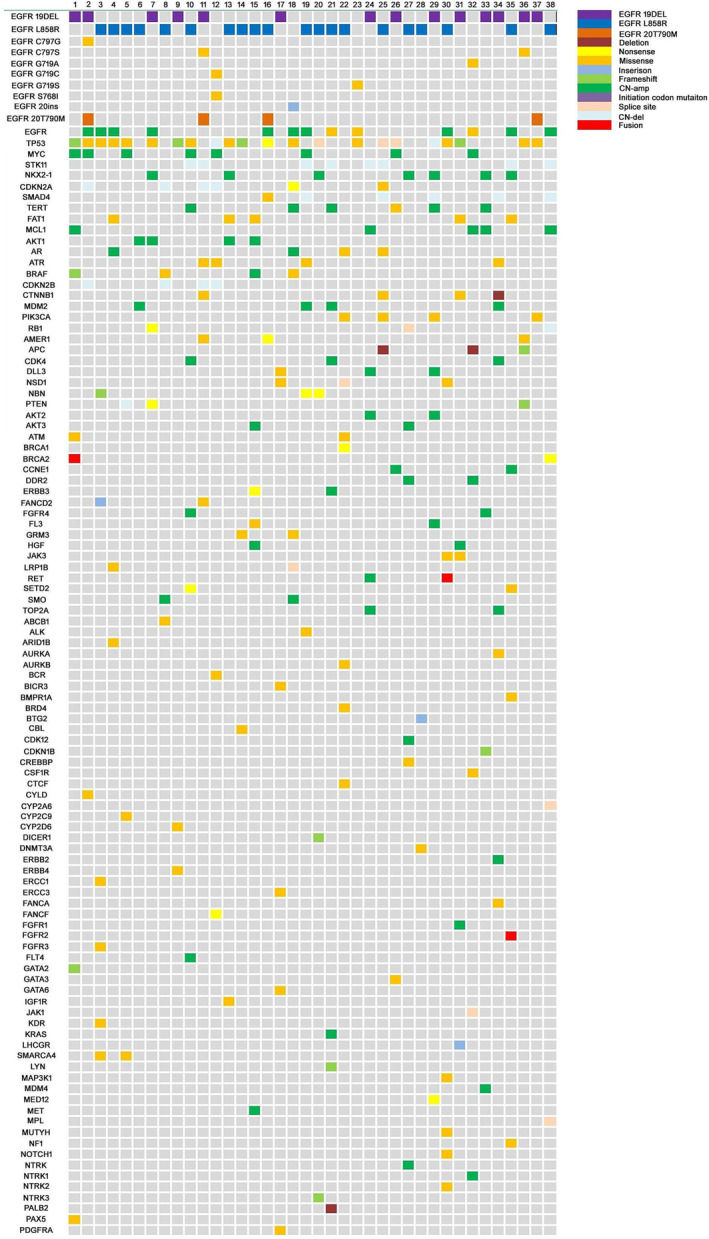
Cerebrospinal fluid genotyping of 38 patients.

EGFR mutations were detected in the CSF cfDNA in all 38 patients, which were consistent with those identified in the primary tumour or plasma of 32 patients. Among the 38 patients (Figure 2), next‐generation sequencing was used to test EGFR in CSF. We found 20 cases of L858R of exon 21 and 14 cases of exon 19 deletion. Other EGFR mutation included EGFR exon 20 insertion (*n* = 1), EGFR G719A of exon 18 (*n* = 1), EGFR G719S of exon 18 (*n* = 1), EGFR S768I of exon 20 and G719C of exon 18 (*n* = 1).

#### 3.2.2 Concurrent genomic alterations seen with patients not received TKI

Among the six EGFR‐mutated LM patients whose CSF was obtained before they received EGFR‐TKIs, TP53 missense mutation (50%, *n* = 3) was the most frequently tested accompanying gene in CSF. Multiple copy number variants (CNV) were mainly found in CSF cfDNA, including copy number amplification of and CDK4 (*n* = 1), EGFR (*n* = 1), and ERBB2 (*n* = 1).

#### 3.2.3 Concurrent genomic alterations seen with patients after first TKI failure

Among the 10 patients whose CSF was obtained after they experienced progression on first TKI, TP53 missense mutation (40%, *n* = 4), EGFR copy number amplification (40%, *n* = 4), NKX2‐1 copy number gain (30.0%, *n* = 3) was detected with high frequency.

**FIGURE 3:**
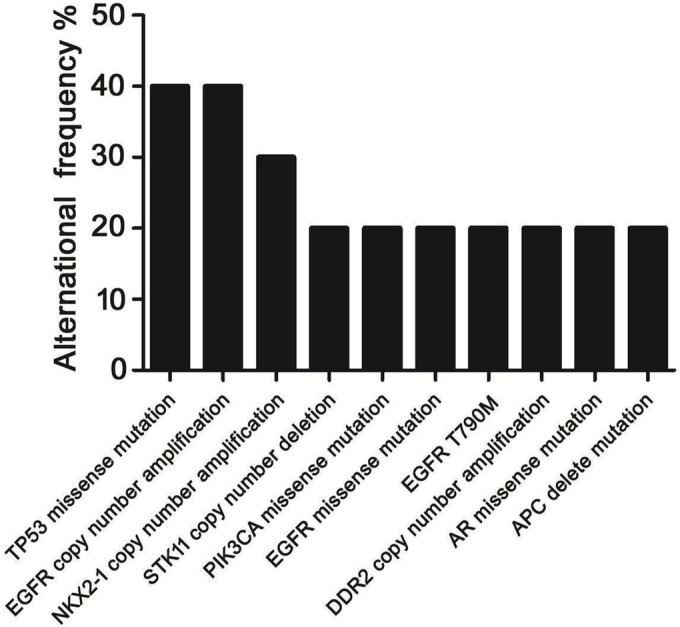
Concurrent genomic alterations seen with patients' progression on first TKI.

T790M mutation was found only in two patients. We also found PIK3CA missense mutation (*n* = 1), FGFR2 gene fusion (*n* = 1), and PALB2 gene deletion (*n* = 1) in CSF cfDNA (Figure 3).

#### 3.2.4 Concurrent genomic alterations seen with patients progression on third TKI

**FIGURE 4:**
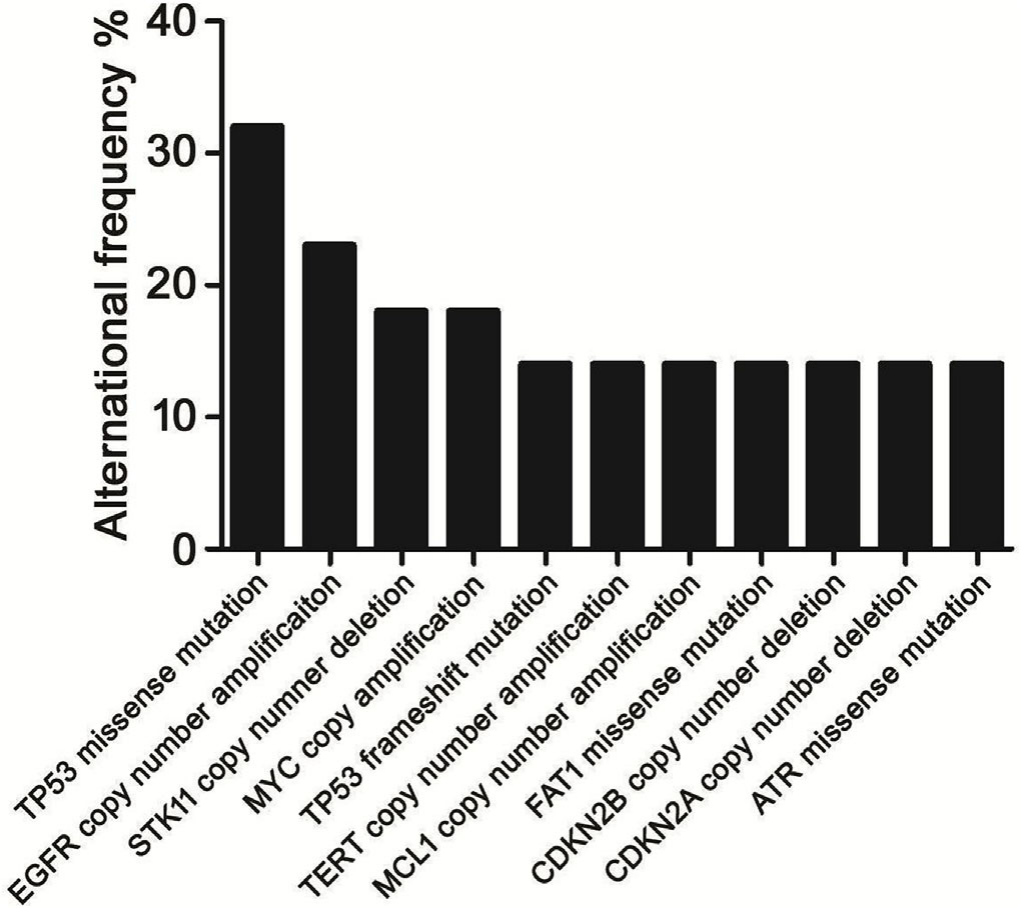
Concurrent genomic alterations seen with patients' progression on third TKI.

Twenty‐two patients were studied with CSF genotyping after they progressed on third EGFR‐TKI (Figure 4). TP53 missense mutation (31.8%, *n* = 7), EGFR amplification (22.7%, *n* = 5), STK11 deletions (18.2%, *n* = 4) and MYC amplification (18.2%, *n* = 4) was detected with high frequency. Two patients harboured T790M mutation. CSF of seven patients was found with known mechanisms of acquired resistance to third EGFR‐TKIs. C797S mutation was found in two patients. T790M was found only in one of these two patients, and the two mutations were in cis. EGFR C797G was found in one patient who was also T790M positive in CSF, and the mutations were in cis. Possible EGFR‐independent resistant mechanism included PIK3CA missense mutation (4.5%, *n* = 1), MET amplification (4.5%, *n* = 1), CDK4 amplification (4.5%, *n* = 1), RET gene fusion (4.5%, *n* = 1). Eight patients with T790M detected by either tumour or plasma before 3rd TKI were included for further analysis. Upon progression, loss of T790M in CSF was seen in all patients.

### 3.3 Tumor mutational burden

**FIGURE 5:**
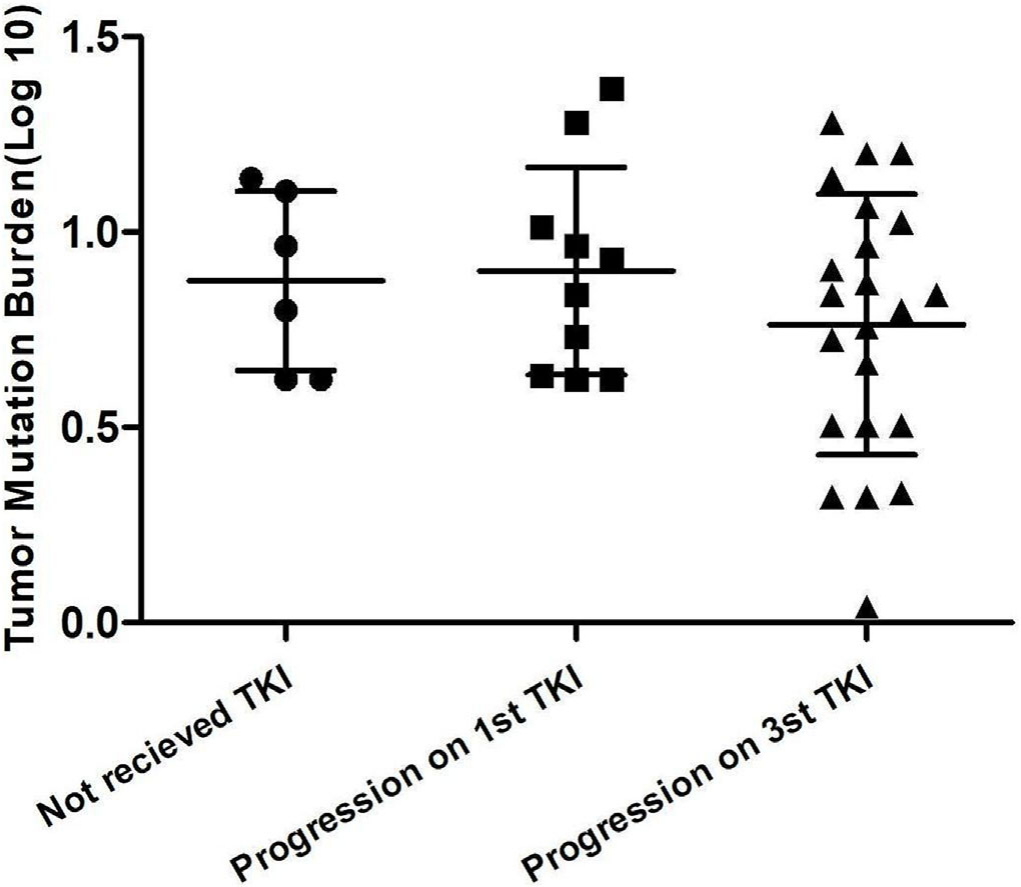
The difference of TMB in CSF among the three groups of patients who had not received TKI, progressed on first TKI and progressed on third TKI.

In the 38 cases, average TMB was 8.4 mutations/Mb (median 6.9, range 1.1–23.83 mutations/Mb); in 12 cases, TMB was more than 10 mutations/Mb. The median TMB of patients who had not received TKI, progressed on first TKI and progressed on 3rd TKI was 8.4 mutations/Mb, 7.9 mutations/Mb and 9.5 mutations/Mb, respectively. No significant difference was found among the three groups (Figure 5). The average TMB of patients harbouring EGFR 19del and L858R was 6.88 mutations/Mb and 9.90 mutations/Mb (*p* > 0.05).

### 3.4 OS and OS_LM_ associated with the concurrent genetic alterations

**FIGURE 6:**
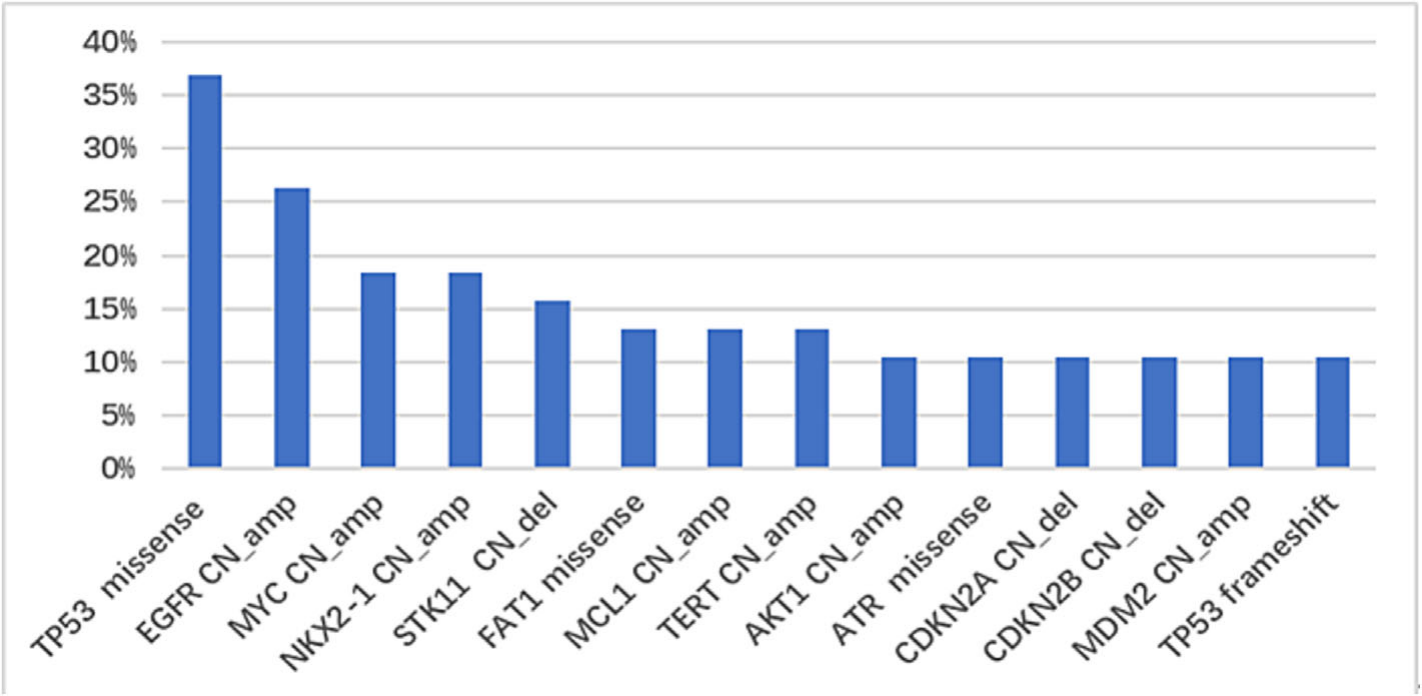
Concurrent genetic alterations of CSF in all patients.

**FIGURE 7:**
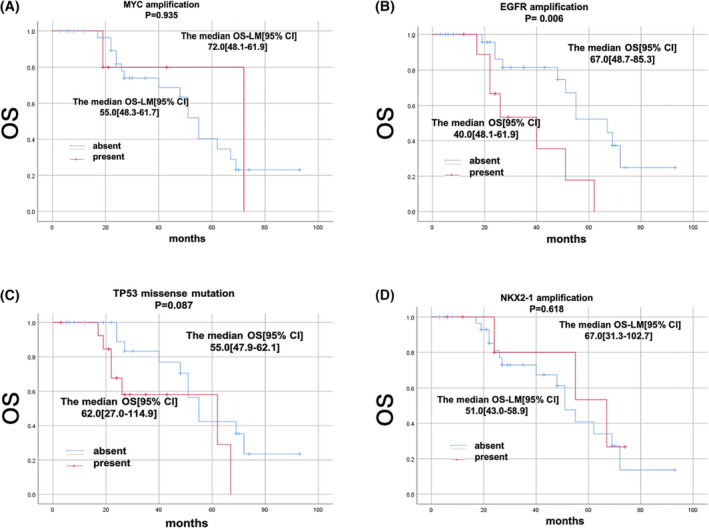
OS associated with the presence or absence of related genes. Kaplan–Meier curves of patients with or without relative genes. (A) With or without co‐mutation of TP53 missense mutation. (B) Presence or absence of EGFR amplification. (C) Presence or absence of MYC amplification. (D) Presence or absence of NKX2‐1 amplification.

**FIGURE 8:**
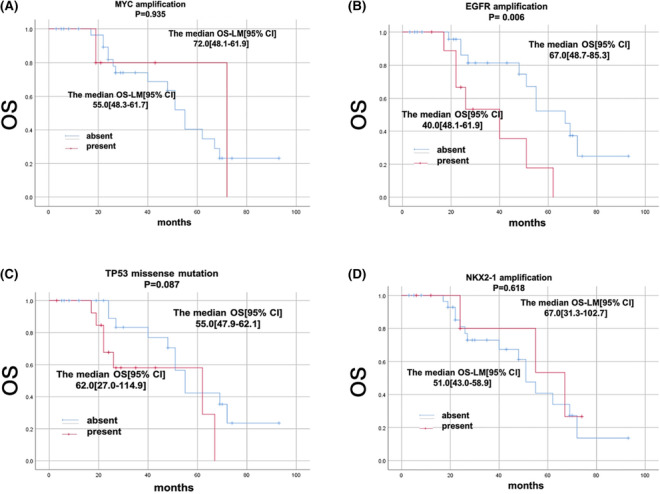
OSLM associated with the presence or absence of related genes. Kaplan–Meier curves of patients with or without relative genes. (A) With or without co‐mutation of TP53 missense mutation. (B) Presence or absence of EGFR amplification. (C) Presence or absence of MYC amplification. (D) Presence or absence of NKX2–1 amplification.

The median OS of all patients was 55 months. In the whole cohort, the median OS_LM_ was 38 months. We analysed concomitant mutations with detected frequency over 10% (Figure 6) to evaluate their impact on OS and OS_LM_. Univariate analysis using Kaplan–Meier method revealed that EGFR amplification was associated with shortened OS. Other concurrent genetic alterations were not significantly associated with shortened OS (Figure 7) or OS_LM_ (Figure 8).

## 4 DISCUSSION

Studies had suggested that CSF is more sensitive than plasma to detect genotyping in NSCLC of LM.^8^, ^10^ Up to now, concurrent molecular alterations that might influence the sensitivity to anticancer treatment and patients' prognosis have been identified.^11^ Concomitant mutations were treated as negative predictive factors of TKI therapy in EGFR‐mutant NSCLC.^12^ In our study, TP53 missense mutation was the most common concurrent mutation in all three groups no matter patients received EGFR TKI or not. However, in the Kaplan–Meier analysis, TP53 missense was not related with shorter OS or OS_LM_. In those patients who did not receive TKI, concurrent genetic alterations of CDK4^8^ and ERBB2^13^ was detected, which may reduce the efficacy of EGFR TKIs, thus suggesting the need to define individualised treatment strategy for LM patients.^13^

Besides TP53 mutation, EGFR amplification was the second frequently concurrent mutated genes in patients received first or third TKI, which was consistent with Professor Wu's report.^8^ In Professor Zhou's study, co‐occurring mutations of TP53 and EGFR were correlated with poor prognosis in patients with leptomeningeal metastases of lung adenocarcinoma.^14^ Our research firstly found EGFR amplification was related with lower OS in LM patients (*p* = 0.006) and slightly lower in OS_LM_ (*p* = 0.08). Maybe it is necessary to further explore whether EGFR amplification participate in the process of the development of meningeal metastasis and whether it is related with the treatment of meningeal metastasis of lung cancer patients.

As for acquired resistance to first TKI in our study, T790M gatekeeper mutation was detected only in two (2/10, 20%) patients' CSF when they progressed on first TKI, which was lower than those in previous studies.^8^, ^15^ EGFR‐T790M mutation of CSF cfDNA was less frequent when compared with extra‐CNS tumours that acquired resistance to EGFR‐TKIs reported before.^16^ As T790M is mediated by TKI exposure, poor TKI penetration into CNS than extra‐CNS tumours is likely to be associated with low incidence of T790M in CNS.^17^ Other acquired resistance mechanism of first TKI such as MET or HER2 amplification^16^, ^18^, ^19^ also not detected CSF in our study. The most frequent acquired resistance to third TKI such as EGFR C797S^11^ has been identified in patient NO.11 and NO.37. Due to the concurrent T790M mutation on the same allele with C797S (in cis), patient NO.11 confers resistance to all generations of EGFR‐TKIs. As for patient NO.37, only C797S mutation was detected in cfDNA; thus, first generation TKIs maybe a strategy to overcome EGFR C797S resistance mutation acquired following osimertinib.^20^ The novel tertiary EGFR C797G mutation concurrent with T790M was found in patient NO.1 in our study, which was reported only in a pleural biopsy specimen of a single patient who progressed on second‐line osimertinib.^21^ Loss of T790M was one of acquired resistance mechanism to osimertinib.^22^ In our study, T790M positive was detected in eight patients by either tumour or plasma when they progressed on first TKI; however, in CSF, it was not detected in all these patients when progressed on third TKI. Given the limited availability of matched pre‐ and post‐osimertinib CSF cfDNA, it need further exploration to verify the existence of loss of T790M in CSF cfDNA in NSCLC of LM. Resistance mechanisms of third TKI like PIK3CA mutation, MET amplification, CDK4 amplification and RET gene fusion^11^, ^22^, ^23^ have been identified in CSF cfDNA in our study. Based on these findings and resistance mechanism, combinatorial approaches may solve the resistance of osimertinib in LM patients.

Up to now, no previous article reports the TMB of lung cancer of leptomeningeal metastasis. The mean TMB of 8.4 mutations/Mb in our study was higher than previous report of 5 mutations/Mb of EGFR mutant CSF.^24^ TMB of 12 patients was more than 10 mutations/Mb. However, in our study, there was no significant difference of TMB among patients received TKI or not. As for EGFR sensitive mutation, TMB of patients harbouring EGFR 21L85R was higher than patients harbouring EGFR 19del, while the difference was not significant. High TMB appeared to be associated with clinical benefit to ICIs.^25^ Although EGFR positive NSCLC patients do not derive a significant benefit from ICIs therapy,^26^ Orient 31 demonstrated sintilimab with bevacizumab plus chemotherapy prolonged PFS (6.9 vs. 4.3 months) in patients with EGFR mutated nonsquamous NSCLC who progressed after EGFR‐TKI therapy than chemotherapy.^27^ As for those LM patients especially those with relatively high TMB, ICIs combined with other agents such as chemotherapy or angiogenesis inhibitor maybe a therapeutic strategy when they progressed on EGFR‐TKI.

The limitations of our study must be acknowledged. Only limited 38 patients were enrolled in this retrospective study as the low incidence of leptomeningeal metastasis. As a retrospective study, we did not get the matched plasma and tissue cfDNA for analysis.

## 5 CONCLUSIONS

EGFR amplification indicated poor prognosis in EGFR‐mutated lung cancer with leptomeningeal metastases, providing a novel pathogenesis and treatment direction of these patients.

## AUTHOR CONTRIBUTIONS

Di Geng and Jinghong Li designed the study and prepared the first draft of the paper. Ruina Nui, Jinghong Li, Sanxing Guo and Qianqian Guo contributed to the acquisition of the data. Siyuan Huang and Yurong Wang were responsible for statistical analysis of the data. Di Geng was responsible for interpretation of data.

## CONFLICT OF INTEREST STATEMENT

All authors disclosed no relevant relationships.

## ETHICS STATEMENT

All the sampling and experimental protocols has been approved by the Research Ethics Committee of the First Affiliated Hospital of Zhengzhou University. All the authors agree to the publication of this paper and the availability of data after publication. Written informed consent was received from all the participants.

## Data Availability

The data that support the findings of this study are available from Di Geng. Restrictions apply to the availability of these data, which were used under license for this study. Data are available from the author(s) with the permission of Di geng.
